# A Cotyledon-Derived *Agrobacterium rhizogenes*-Mediated Transformation System for Regenerating Transgenic ‘Carrizo’ Citrange Plants

**DOI:** 10.3390/plants15213410

**Published:** 2026-11-02

**Authors:** Qihuan Deng, Chao Yang, Haoning He, Mingyan Wang, Xiaochun Wang, Qiying Wang, Xuefeng Wang, Yaobin Lv, Xinyuan Wu, Jun He, Changyong Zhou

**Affiliations:** 1 Integrative Science Center of Germplasm Creation in Western China (CHONGQING) Science City, National Citrus Engineering Research Center, Citrus Research Institute, Southwest University, Chongqing 400712, China; 2 Xianghu Laboratory, Hangzhou 311258, China13375001682@163.com (X.W.)

**Keywords:** *Agrobacterium rhizogenes*, hairy root transformation, citrus, ‘Carrizo’ citrange, cotyledon explant, plant regeneration, genetic transformation

## Abstract

The citrus genetic transformation system is essential for genetic improvement and functional studies of the crop. *Agrobacterium rhizogenes*-mediated transformation in citrus has enabled efficient and rapid generation of transformed root tissues compared to conventional *A. tumefaciens*-mediated transformation. However, suitable explants and transformation conditions in this system still require further optimization to obtain ample clean transformed roots, which is a prerequisite for subsequent shoot regeneration when stable transgenic plants are desired. In this study, we established an integrated cotyledon-derived, *A. rhizogenes*-mediated hairy root transformation and plant regeneration system for ‘Carrizo’ citrange. Both cotyledon and epicotyl explants yielded transformed hairy roots; however, cotyledon explants exhibited higher hairy root induction and hairy root transformation efficiency under their respective optimized conditions. A 3 h AS pre-induction time, a 30 min infection time, and a 2,4-D concentration of 0.1 mg/L were identified as the optimal conditions for cotyledon transformation, and the hairy root induction rate, GFP-positive hairy root frequency, and hairy root transformation efficiency reached an average value of 69.22 ± 3.13%, 74.21 ± 1.08%, and 51.36 ± 1.99%, respectively. Transformed hairy roots were further regenerated into shoots via a callus-mediated regeneration pathway, followed by micrografting and subsequent grafting to recover intact plants. Finally, regenerated plants were successfully obtained and molecularly validated. This study reports a highly efficient *A. rhizogenes*-mediated transformation and regeneration system for citrus.

## 1. Introduction

Citrus is one of the most economically important fruit crops worldwide, contributing substantially to the global fruit industry [1]. Conventional breeding of citrus is largely constrained by its long juvenile phase, perennial growth habit, and complex genetic background, which limit the rapid improvement of important agronomic traits [2,3]. Therefore, efficient genetic transformation technologies are essential for directly introducing desirable traits and enabling genome editing-based crop improvement. Despite significant advances in citrus biotechnology, the establishment of stable and efficient transformation systems remains challenging in citrus [3,4].

Conventional citrus transformation systems are mainly based on *Agrobacterium tumefaciens*-mediated transformation, which has enabled the generation of transgenic plants in various citrus genotypes, including ‘Carrizo’ citrange [5], and facilitated functional studies of genes associated with important agronomic traits [6,7], which have been constrained by genotype-dependent responses, regeneration capacity, and tissue culture constraints [3,4]. Alternatively, *A. rhizogenes*-mediated hairy root transformation has emerged as a valuable approach for plant genetic studies. Through Ri plasmid-mediated T-DNA transfer, *A. rhizogenes* induces genetically transformed hairy roots that can be rapidly generated and used for genetic studies [6,7]. Compared with conventional whole-plant transformation approaches, hairy root systems provide a rapid and efficient platform for obtaining genetically modified tissues and have been successfully applied in diverse plant species, including citrus [8]. However, these systems are generally limited to transformed root tissues, which restricts their application when gene functions need to be evaluated at the whole-plant level. Irigoyen et al. established a citrus hairy root platform for high-throughput identification of antimicrobials against *Candidatus* Liberibacter spp., demonstrating the potential of hairy roots for host–pathogen interaction studies [9]. Subsequently, Ramasamy et al. developed a regeneration strategy from transformed hairy roots and obtained stable transgenic citrus lines [10]. However, the production of sufficient transformed hairy roots and the reproducibility of shoot regeneration remain limiting factors, highlighting the requirement for further optimization of *A. rhizogenes*-mediated citrus transformation systems.

Explant selection and transformation conditions are critical factors influencing the efficiency of *A. rhizogenes*-mediated transformation systems [10,11]. In citrus, different explant sources have been used for *A. rhizogenes*-mediated hairy root induction and regeneration. Irigoyen et al. used single-node budwood tissues from mature citrus plants, while Ramasamy et al. employed tissue culture-derived epicotyl explants and healthy citrus buds for hairy root induction and plant regeneration [9,10]. Since explant developmental status and cellular competence are important factors influencing hairy root transformation efficiency and regeneration capacity, exploring alternative explant sources may provide new opportunities for improving citrus transformation systems [12]. Cotyledon explants have been reported as suitable materials for *A. rhizogenes*-mediated transformation and plant regeneration, suggesting their potential for establishing alternative transformation systems. In addition, cotyledon-based hairy root induction systems have been successfully developed in several plant species, including soybean, pepper, and cotton, demonstrating the strong regenerative competence and transformation potential of juvenile cotyledon tissues [13,14,15]. Therefore, identification of competent explant sources, particularly juvenile explants with high regenerative potential, is important for improving the efficiency and reproducibility of *A. rhizogenes*-mediated transformation systems.

In addition to explant selection, transformation conditions are also critical factors affecting the efficiency of *A. rhizogenes*-mediated transformation systems. The efficiency of T-DNA transfer is influenced by multiple parameters, including acetosyringone (AS) pre-induction time, infection time, and 2,4-dichlorophenoxyacetic acid (2,4-D) concentration. Optimization of infection conditions and culture parameters is essential for improving hairy root transformation efficiency while maintaining explant viability during *A. rhizogenes*-mediated transformation [16,17].

In citrus, *A. rhizogenes*-mediated hairy root systems have enabled rapid functional analyses, including gene overexpression, genome editing, and molecular characterization [11,17]. When gene functions are related to aerial tissues, developmental traits, metabolite production, disease resistance, or plant–environment interactions, stable transgenic plants are still required for whole-plant functional evaluation. Therefore, recovery of regenerated shoots from transformed hairy roots represents an important step toward extending the application of *A. rhizogenes*-mediated transformation beyond root-based analyses. Although Ramasamy et al. demonstrated whole-plant recovery from transformed citrus hairy roots, further optimization of regeneration media, hormone combinations, and hairy root material selection is needed to improve the efficiency and reproducibility of this process [10].

Building on the previously reported *A. rhizogenes*-mediated citrus transformation and regeneration system [10], this study aimed to establish and optimize an efficient, rapid, and straightforward citrus transformation system as an alternative to conventional approaches, using cotyledon explants of ‘Carrizo’ citrange and linking hairy root transformation with whole-plant recovery. In this study, we established an integrated cotyledon-based *A. rhizogenes*-mediated hairy root transformation and regeneration pipeline in ‘Carrizo’ citrange. This system combines the rapid generation of transformed hairy roots with subsequent recovery of stable transgenic plants, providing an alternative strategy for citrus functional studies beyond conventional transformation approaches. The contribution of this study lies in expanding the explant options for the existing citrus hairy root transformation and regeneration system by establishing a cotyledon-based workflow. We used cotyledons as explants as an alternative to epicotyls and optimized the key transformation parameters, including AS pre-induction time, infection time, and 2,4-D concentration, to improve hairy root transformation efficiency. Furthermore, a regeneration strategy combining callus induction, shoot regeneration, micrografting, and plant recovery was established to obtain stable transgenic citrus plants.

## 2. Results

### 2.1. Establishment and Optimization of a Cotyledon-Based A. rhizogenes-Mediated Hairy Root Transformation System in ‘Carrizo’ Citrange

**Figure 1:**
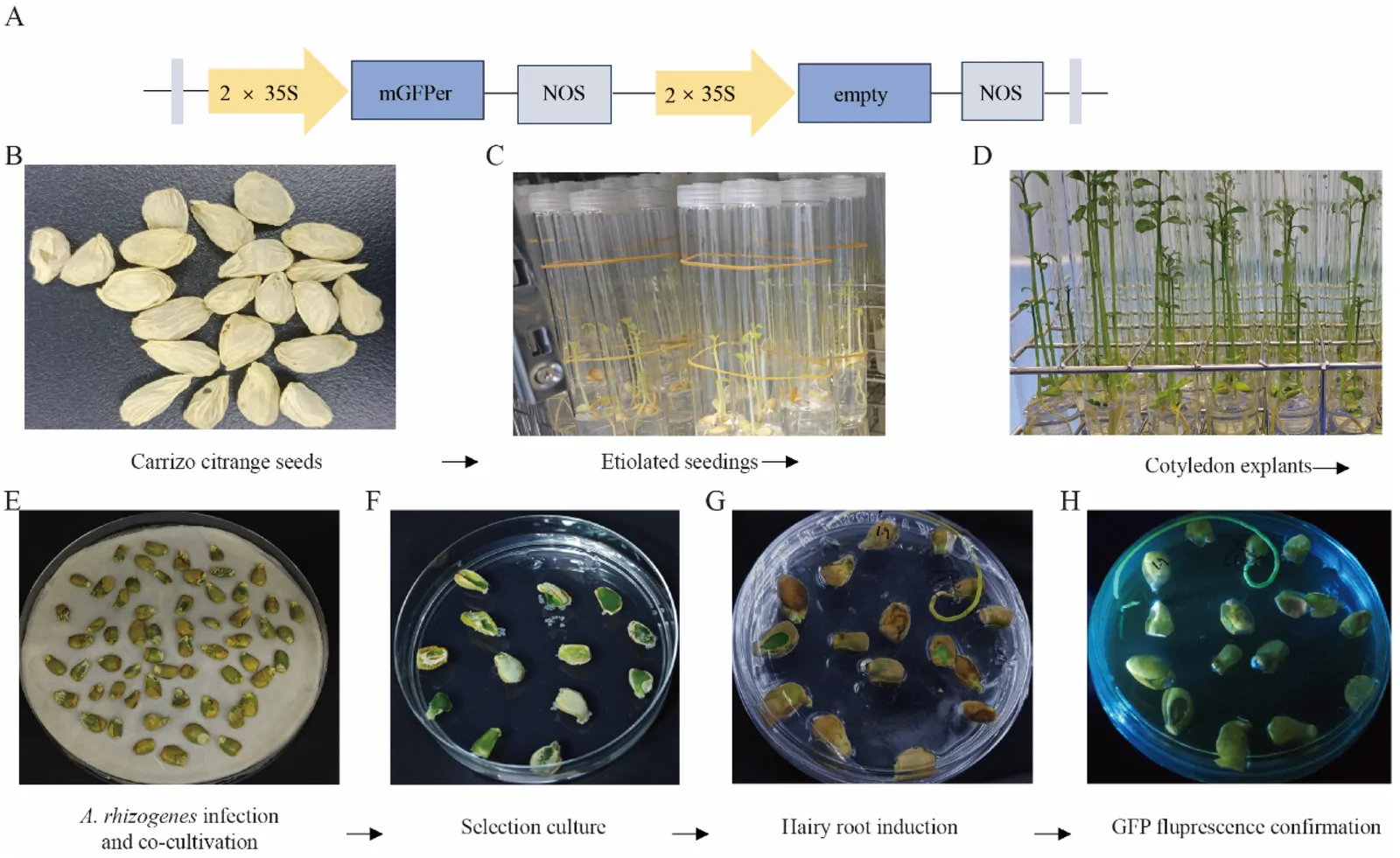
Establishment of a cotyledon-based *Agrobacterium rhizogenes*-mediated hairy root induction system in ‘Carrizo’ citrange. (**A**) Schematic view of the GFP-containing empty-vector construct. (**B**–**H**) Representative images showing ‘Carrizo’ citrange seed germination, etiolated seedling preparation, cotyledon explant preparation, *A. rhizogenes* infection and co-cultivation, selection culture, hairy root induction, and GFP fluorescence confirmation.

To facilitate the identification of transformed tissues, GFP-based visual screening was incorporated into the transformation system. The binary vector pNmGFPer containing a GFP reporter gene driven by a double CaMV35S promoter was used as the empty-vector control, and its vector structure is shown in Figure 1A. GFP fluorescence enabled rapid identification and selection of positive transformation events during hairy root induction and regeneration. The overall workflow of the cotyledon-based *A. rhizogenes*-mediated hairy root induction system, including seedling preparation, cotyledon explant preparation, bacterial infection, co-cultivation, and selection of transformed materials, is illustrated in Figure 1B–H. A one-factor-at-a-time optimization strategy was adopted to evaluate AS pre-induction time, infection time, and 2,4-D concentration, with the remaining two parameters maintained at fixed levels during the evaluation of each factor. Each treatment was performed using three independent replicates, and hairy root induction rate, GFP-positive hairy root frequency, and hairy root transformation efficiency were subsequently evaluated.

**Figure 2:**
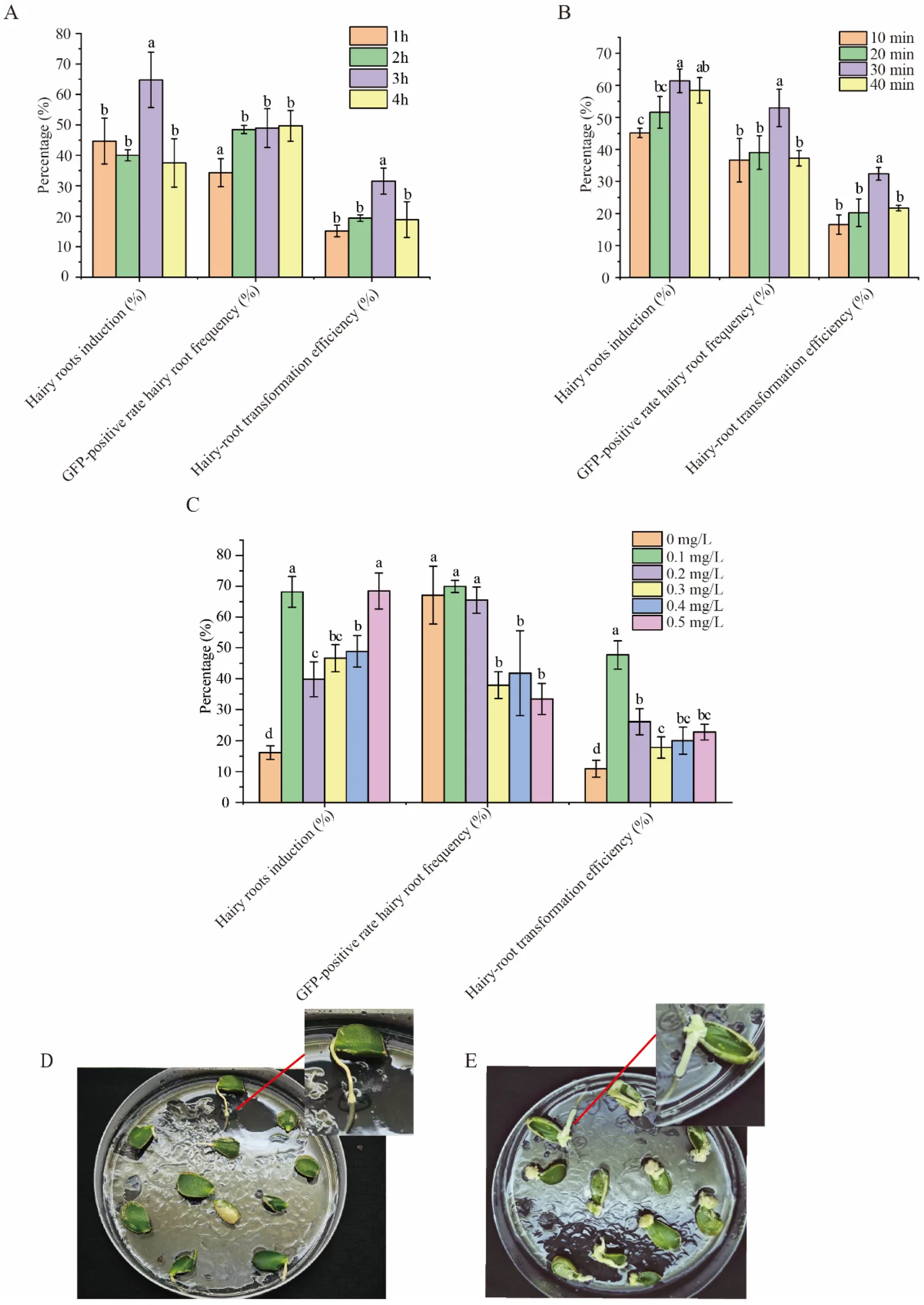
Effects of different transformation conditions on hairy root induction, GFP-positive rate, and hairy root transformation efficiency in Carrizo citrange. (**A**) Effect of acetosyringone (AS) induction time (1–4 h). (**B**) Effect of *A. rhizogenes* infection time (10–40 min). (**C**) Effect of different 2,4-dichlorophenoxyacetic acid (2,4-D) concentrations (0.1–0.5 mg/L). (**D**) Balanced hairy root formation under 0.1 mg/L 2,4-D treatment. (**E**) Excessive callus proliferation induced by 0.5 mg/L 2,4-D. Data are presented as the mean ± SD of three independent replicates. Different lowercase letters above the bars indicate significant differences among treatments within the same parameter according to one-way ANOVA followed by Duncan’s multiple range test (*p* < 0.05).

The AS pre-induction time significantly affected transformation efficiency in cotyledon explants (Figure 2A; Table S1). Different AS pre-induction times (1, 2, 3 and 4 h) were evaluated, while the infection time and 2,4-D concentration were fixed at 30 min and 0.5 mg/L, respectively. Extending the AS pre-induction duration from 1 h to 3 h increased hairy root induction rate from 44.68 ± 7.55% to 64.77 ± 9.05%, GFP-positive hairy root frequency from 34.30 ± 4.60% to 48.95 ± 6.38%, and hairy root transformation efficiency from 15.16 ± 1.91% to 31.54 ± 4.27%, respectively, while further extending the AS pre-induction time to 4 h decreased hairy root induction rate and transformation efficiency, whereas GFP-positive hairy root frequency increased slightly to 49.66 ± 5.06%.

The infection time significantly affected hairy root transformation efficiency (Figure 2B; Table S2). Different infection times (10, 20, and 30 min) were evaluated while the AS pre-induction time and 2,4-D concentration were fixed at 3 h and 0.5 mg/L, respectively. Although extending the infection time from 10 to 20 min showed an increasing trend in hairy root transformation efficiency, the difference was not statistically significant. Compared with 10 min, a 30-min infection time significantly increased the hairy root induction rate, GFP-positive hairy root frequency, and transformation efficiency, reaching 61.41 ± 3.65%, 52.98 ± 5.84% and 32.40 ± 1.97%, respectively. Further extension of the infection time did not further improve hairy root transformation efficiency.

The 2,4-D concentration significantly affected hairy root induction and hairy root transformation efficiency (Figure 2C; Table S3). Different concentrations of 2,4-D (0, 0.1, 0.2, 0.3, 0.4, and 0.5 mg/L) were evaluated under the optimized AS pre-induction time and infection time. In the absence of 2,4-D, hairy root induction was limited, with a hairy root transformation efficiency of only 10.92 ± 2.74%. Supplementation with 2,4-D significantly improved hairy root production, and the highest hairy root transformation efficiency was obtained at 0.1 mg/L 2,4-D, reaching 47.73 ± 4.60%, with a hairy root induction rate of 68.17 ± 5.01% and a GFP-positive hairy root frequency of 69.95 ± 1.98%. Higher concentrations of 2,4-D (0.2–0.5 mg/L) did not further improve hairy root transformation efficiency. Although 0.2 mg/L 2,4-D maintained a relatively high GFP-positive hairy root frequency, both hairy root induction rate and hairy root transformation efficiency were reduced compared with 0.1 mg/L. Moreover, excessive 2,4-D concentrations (0.3–0.5 mg/L) promoted callus accumulation and negatively affected hairy root development, particularly at 0.5 mg/L (Figure 2D–E). Therefore, 0.1 mg/L 2,4-D was selected as the optimal concentration for cotyledon transformation. Combined with the optimized AS pre-induction time (3 h) and infection time (30 min), this condition provided sufficient GFP-positive hairy root materials for subsequent regeneration experiments.

### 2.2. Evaluation of Epicotyl and Cotyledon Explants for Hairy Root Induction and Transformation in ‘Carrizo’ Citrange

The transformation performance of the newly established cotyledon-based system was evaluated in comparison with the previously reported epicotyl-based *A. rhizogenes*-mediated transformation system [10]. However, when epicotyl explants were subjected to an AS pre-induction of 3 h and an infection time of 30 min, excessive bacterial proliferation occurred during selection culture, and repeated antibiotic treatments failed to completely suppress bacterial overgrowth, preventing the stable recovery of transformed hairy roots. Therefore, epicotyl explants were subsequently transformed exactly according to previously reported citrus hairy root transformation conditions. The transformation performance of cotyledon and epicotyl explants was subsequently compared under their respective optimized conditions (Table 1). Cotyledon explants exhibited significantly higher hairy root induction rate, GFP-positive hairy root frequency, and hairy root transformation efficiency than epicotyl explants, with values reaching 69.22 ± 3.13%, 74.21 ± 1.08%, and 51.36 ± 1.99%, respectively. In contrast, epicotyl explants showed substantially lower values, with hairy root induction rate, GFP-positive hairy root frequency, and hairy root transformation efficiency of 14.92 ± 3.22%, 60.67 ± 6.54%, and 8.93 ± 1.29%, respectively. These values represent the mean ± standard deviation (SD) of three independent replicates (*n* = 3). Cotyledon explants showed faster hairy root emergence than epicotyl explants under their respective optimized conditions (Table 1).

### 2.3. Regeneration and Recovery of Stable Transgenic ‘Carrizo’ Citrange Plants from Transformed Hairy Roots

**Figure 3:**
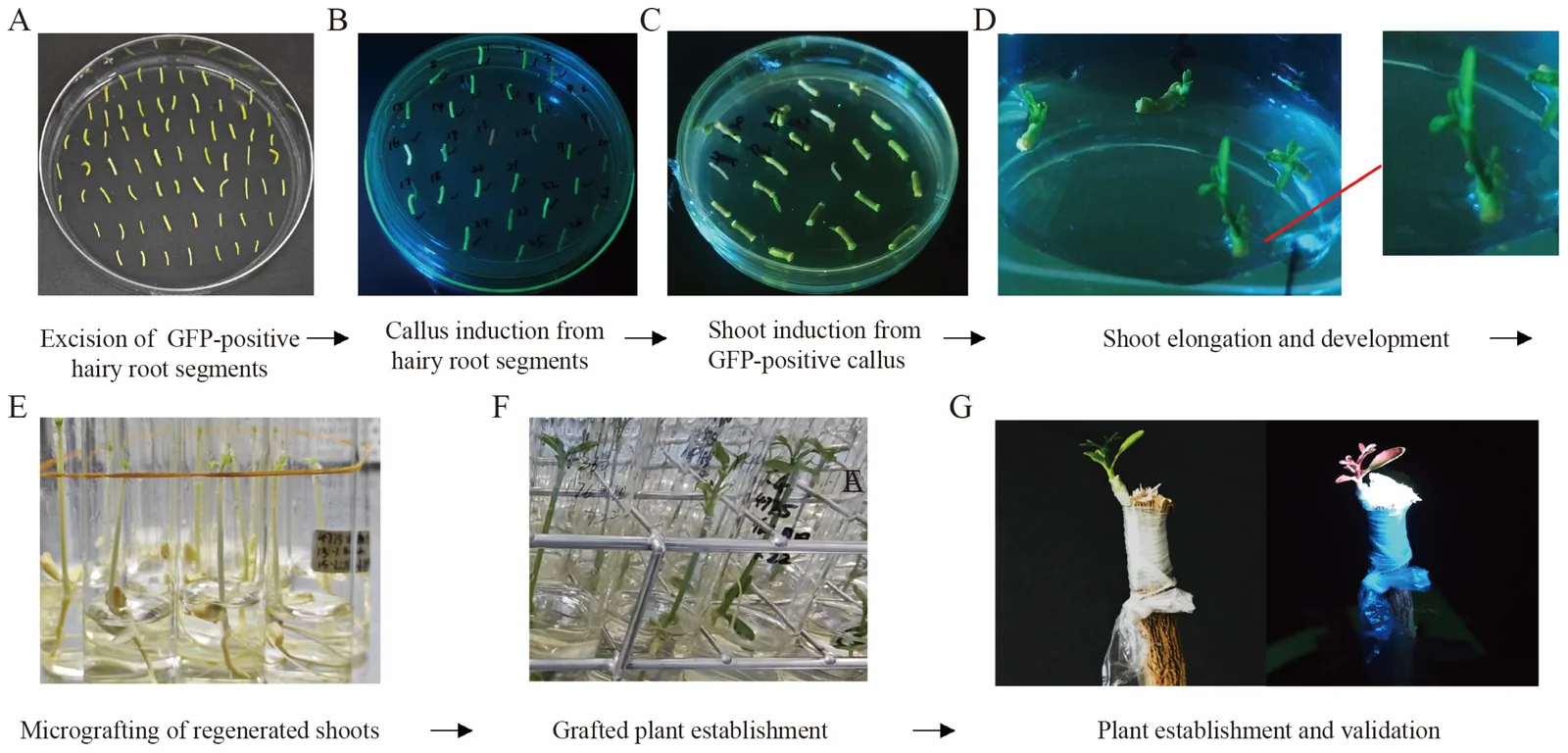
Regeneration and establishment of plants from GFP-positive hairy roots in ‘Carrizo’ citrange. (**A**–**G**) Representative images showing GFP-positive hairy root selection, root segment preparation, callus induction and GFP-positive callus formation, shoot regeneration and elongation, micrografting of regenerated shoots, and establishment of regenerated plants after grafting.

GFP-positive and vigorously growing hairy roots were selected as starting materials for regeneration experiments. Approximately 60 hairy root segments were excised and subjected to an initial callus induction medium to promote callus formation. After 7~10 days of in vitro culture, approximately 30% of hairy root segments exhibited visible swelling and callus formation. The GFP-positive callus tissues were subsequently transferred to regeneration medium to induce adventitious shoot formation. After approximately two weeks of culture, GFP-positive callus tissues derived from hairy root segments were obtained and subsequently transferred to shoot induction medium for adventitious shoot regeneration (Figure 3C,D). Regenerated shoots with expanded leaves were further transferred to shoot elongation medium until reaching a suitable size for grafting (Figure 3E). A total of 14 regenerated shoots were successfully recovered through micrografting onto juvenile rootstocks, followed by subsequent grafting onto mature rootstocks for plant establishment (Figure 3F,G). Following acclimatization and GFP fluorescence screening, six independent transgenic lines and four empty-vector control plants were successfully generated. These results demonstrated that the established pipeline enabled the recovery of regenerated plants from *A. rhizogenes*-induced hairy roots.

### 2.4. Molecular Validation of Regenerated Transgenic Plants

**Figure 4:**
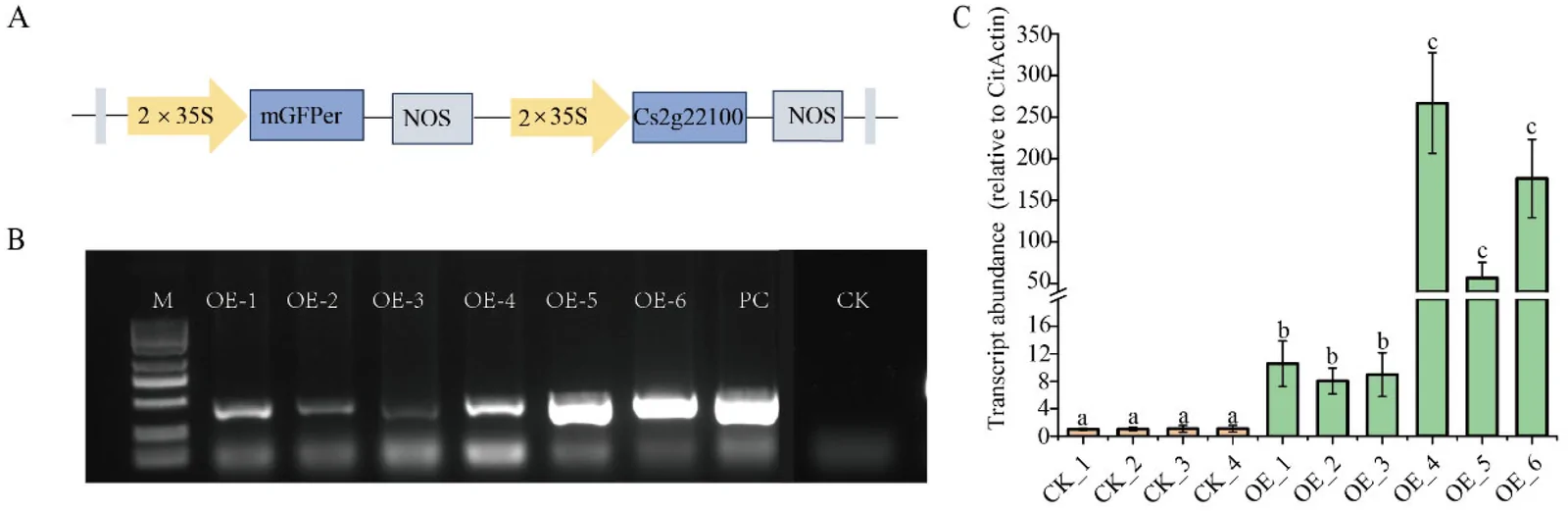
Generation and molecular characterization of *Cs2g22100*-overexpressing ‘Carrizo’ citrange plants. (**A**) Schematic view of the *Cs2g22100* overexpression vector. (**B**) PCR confirmation of *Cs2g22100* integration in transgenic ‘Carrizo’ citrange lines. (**C**) Relative expression levels of *Cs2g22100* in transgenic ‘Carrizo’ citrange lines determined by qRT-PCR. Data in (**C**) represent means ± SD; different lowercase letters indicate statistically significant differences (*p* < 0.05) among groups based on Duncan’s multiple range tests.

To test whether our system can deliver and express a citrus gene of interest in addition to the GFP marker, the sweet orange gene *Cs2g22100* was introduced into the same vector backbone under the control of an independent CaMV35S promoter (Figure 4A). Following hairy root transformation, shoot regeneration and two rounds of grafting, regenerated plants were obtained for molecular validation. Molecular analyses were performed to confirm the presence of the *Cs2g22100* expression cassette and the expression of the target gene in regenerated plants. PCR analysis detected the presence of the *Cs2g22100* expression cassette in regenerated plants, whereas no corresponding amplification was observed in empty-vector plants (Figure 4B).

After successful graft establishment on three-year-old rootstocks, *Cs2g22100* expression levels were evaluated by quantitative real-time PCR. We found that the relative *Cs2g22100* expression levels in OE_1 to OE_6 lines were elevated to 10.58-, 8.04-, 8.96-, 266.73-, 56.63-, and 176.04-fold compared with empty-vector plants, respectively (Figure 4C). Together, these results verified the successful generation of stable *Cs2g22100*-overexpressing ‘Carrizo’ citrange plants through the established *A. rhizogenes*-mediated transformation and regeneration pipeline. These findings extend the GFP-based validation by demonstrating the applicability of the system to a citrus gene-of-interest overexpression construct, providing plant materials for subsequent functional characterization.

## 3. Discussion

### 3.1. Cotyledon and Epicotyl Provide Complementary Sources for A. rhizogenes-Mediated Hairy Root Production in ‘Carrizo’ Citrange

The explant type is a critical factor influencing the efficiency and reproducibility of *A. rhizogenes*-mediated transformation systems. Different explant types exhibit distinct responses to *Agrobacterium* infection, cellular dedifferentiation, and subsequent regeneration processes. In citrus, epicotyl explants are widely used for genetic transformation because of their juvenile characteristics and regenerative potential [3,4]; however, transformation performance remains highly dependent on genotype background, bacterial interaction, and culture conditions [3,6,7].

In this study, both epicotyl and cotyledon explants were evaluated as sources for *A. rhizogenes*-mediated hairy root induction in ‘Carrizo’ citrange. Although epicotyls have been commonly used in citrus transformation systems, their response under the tested conditions was limited by excessive bacterial proliferation and insufficient recovery of transformed hairy root materials. Therefore, cotyledon explants were investigated as an additional explant source rather than a replacement for the conventional epicotyl-based system.

Cotyledon tissues have been successfully applied for *A. rhizogenes*-mediated transformation in several plant species and have shown strong potential for hairy root induction and subsequent genetic analyses [15,18,19,20]. Their enhanced responsiveness may be associated with their juvenile developmental status and cellular plasticity, which facilitate tissue reprogramming during in vitro culture [21,22]. In this study, the cotyledon-based system provided an efficient source of GFP-positive hairy roots, while the combination of cotyledon and epicotyl explants increased the availability of transformed root materials for subsequent regeneration experiments. These findings demonstrate that expanding explant sources represents an effective strategy for improving the flexibility and efficiency of citrus hairy root transformation systems. Cotyledons were used as the explant source for system establishment due to their juvenile developmental status and potential regenerative competence [18]. Cotyledons and epicotyls, the latter representing seedling stem tissues, were readily available from aseptically grown seedlings and were therefore selected for establishing the in vitro workflow. Leaf and root segments were not evaluated as starting explants in this study, and their suitability remains to be assessed. Based on previous studies showing that AS pre-induction time, infection time, and auxin supplementation influence *A. rhizogenes*-mediated transformation efficiency and explant responses [7,19,23], these three factors were selected for optimization of the cotyledon-based transformation system in ‘Carrizo’ citrange.

### 3.2. Optimization of Infection and Culture Conditions Improves the Production of Regeneration-Competent Hairy Roots

Our results indicate that the optimization of transformation parameters significantly improved the production of transformed hairy root materials in ‘Carrizo’ citrange cotyledons. AS is a phenolic compound released from wounded plant tissues that activates *Agrobacterium* virulence genes and promotes T-DNA transfer [24,25]. In this study, extending AS pre-induction time from 1 h to 3 h significantly increased hairy root transformation efficiency, suggesting that sufficient AS exposure enhanced bacterial virulence activation and improved T-DNA transfer. However, further extension of the AS pre-induction time did not provide additional benefits, indicating that prolonged AS exposure may not further enhance hairy root transformation efficiency under the established conditions.

Infection time is another critical factor affecting *A. rhizogenes*-mediated transformation [16,19]. Previous studies have demonstrated that infection time influences hairy root transformation efficiency by affecting the interaction between bacteria and plant tissues during T-DNA delivery [19]. In the present study, a 30 min infection time resulted in the highest values for the evaluated transformation indices in cotyledon explants, suggesting that this duration was optimal under the established conditions.

The concentration of 2,4-D markedly affected the transformation performance and developmental responses of cotyledon explants. Auxins, including 2,4-D, regulate cell division, dedifferentiation, and callus formation during in vitro culture [26]. However, their effects are concentration-dependent, and excessive auxin levels may disrupt normal tissue development and reduce regeneration capacity [27]. In this study, compared with the treatment without 2,4-D, supplementation with an appropriate concentration of 2,4-D improved the evaluated transformation indices and increased the recovery of GFP-positive hairy roots. In contrast, higher 2,4-D concentrations promoted excessive callus formation and tissue browning or necrosis, thereby inhibiting normal hairy root development and reducing transformation performance. These results indicate that an appropriate 2,4-D concentration promotes hairy root induction while maintaining normal tissue development.

Collectively, these findings demonstrate that optimization of AS pre-induction time, infection time, and 2,4-D concentration is essential for improving transformation performance and generating sufficient GFP-positive hairy root materials for subsequent regeneration in ‘Carrizo’ citrange.

### 3.3. Callus-Mediated Regeneration Enables the Recovery of Stable Transgenic Citrus Plants from Transformed Hairy Roots

The regeneration of stable transgenic plants from transformed hairy roots represents a critical step in extending *A. rhizogenes*-mediated transformation from root-level functional analysis to whole-plant studies. Existing citrus hairy root systems have mainly been used for rapid gene functional analysis in transformed root tissues [11,17]. Although the regeneration of complete plants from transformed hairy roots has been reported in citrus, cotton, and other fruit-tree species [14,18,28], the root-to-shoot transition remains technically demanding, particularly in woody plants with limited regeneration competence [29,30].

In the present study, the evaluation of explant types and optimization of AS pre-induction time, infection time, and 2,4-D concentration, together with GFP-based screening, yielded sufficient GFP-positive hairy root materials for subsequent regeneration. To improve shoot regeneration from transformed hairy roots, a short pre-regeneration culture stage was introduced before shoot induction. During this stage, hairy root segments underwent swelling and callus formation, enabling subsequent shoot regeneration. Callus formation represents an important transitional state during indirect regeneration, in which differentiated cells undergo developmental reprogramming and acquire the ability to regenerate shoots [21,22]. Accordingly, the transient callus phase observed in this study may have facilitated the transition from transformed hairy root tissues to shoot regeneration [31]. Direct shoot regeneration from hairy roots without an intervening callus phase was not systematically evaluated in this study. Although the callus-mediated procedure enabled plant regeneration, the possibility of somaclonal variation remains, and the genetic fidelity of regenerated plants requires further assessment. However, PCR-based detection of the expression cassette alone cannot distinguish T-DNA integrated into the plant genome from *Agrobacterium*-derived DNA; therefore, residual *Agrobacterium* DNA cannot be fully excluded. Future studies could screen genomic DNA from regenerated plant tissues by PCR using validated primers targeting an *Agrobacterium* chromosomal sequence, together with appropriate controls. A positive result would indicate the presence of *Agrobacterium* DNA in the sample, whereas a negative result would indicate that it was not detected under the assay conditions.

Following shoot regeneration, established grafting procedures commonly used in citrus transformation were applied to promote the recovery and continued growth of regenerated shoot [7,32,33]. The regenerated shoots were first micrografted onto etiolated citrus seedlings and subsequently regrafted onto established soil-grown rootstocks, ultimately yielding stable *Cs2g22100*-overexpressing plants. Therefore, the introduction of a pre-callus culture stage facilitated shoot regeneration from transformed hairy roots, whereas established grafting procedures facilitated the subsequent recovery of complete plants. These findings indicate that GFP-positive hairy roots can serve not only as materials for root-level functional analysis but also as starting tissues for the recovery of stable transgenic citrus plants.

Compared with conventional *Agrobacterium tumefaciens*-mediated transformation systems, the established *A. rhizogenes*-mediated pipeline provides an alternative strategy by combining rapid generation of transformed tissues with subsequent recovery of transgenic plants. However, this workflow involves multiple culture and regeneration steps- optimization of the cotyledon-based system improved the production of GFP-positive hairy roots under in vitro conditions, providing more starting materials for subsequent shoot regeneration. When coupled with a reproducible shoot regeneration procedure and further GFP screening, this could increase the number of positive plants recovered per batch and reduce the number of transformation batches needed to obtain sufficient experimental materials. These potential benefits in plant recovery and total time requirements remain to be quantified through direct comparisons with conventional methods. Unlike conventional hairy root systems that are primarily limited to root-associated analyses, the regeneration strategy developed in this study extends the application of hairy root transformation to whole-plant functional studies. This could allow candidate genes to be initially evaluated in hairy roots and subsequently investigated in regenerated plants, providing opportunities to compare gene-associated responses at the root and whole-plant levels. The *Cs2g22100* experiment provides a proof of concept for introducing and expressing a citrus gene of interest through this workflow; its biological function remains to be characterized. Therefore, this platform may facilitate future studies involving gene function characterization, genome editing, and molecular breeding in citrus.

## 4. Materials and Methods

### 4.1. Plant Materials and Growth Conditions

Seeds of ‘Carrizo’ citrange (*Citrus sinensis* × *Poncirus trifoliata*) were used as the primary plant material in this study. Seeds were obtained from commercial orchards located in Zhongxian County, Chongqing, China. The outer and inner seed coats were manually removed, and seeds were surface sterilized with 5% (*w*/*v*) sodium dichloroisocyanurate solution (Macklin, Shanghai, China) for 15 min with intermittent agitation [34]. Sterilized seeds were rinsed three times with sterile distilled water and cultured on modified MS germination medium (Coolaber, PM10121-307-50 × 0.5 L, Beijing, China). Seedlings were maintained in darkness at 28 ± 2 °C for 3 weeks to obtain etiolated seedlings and subsequently transferred to a 16 h light/8 h dark photoperiod for 13 days before explant preparation. The compositions of all media used in this study are listed in Table 2.

### 4.2. Vector Construction and Preparation of A. rhizogenes

To assess whether the system could support the introduction and expression of a citrus gene of interest in addition to the GFP reporter, *Cs2g22100* was selected for proof-of-concept evaluation. This candidate terpene synthase gene was selected based on our preliminary expression analysis, which suggested that its expression responds to Huanglongbing infection, with the aim of generating transgenic plants for subsequent functional characterization. Two binary vectors were used in this study. The empty-vector pNmGFPer containing a GFP reporter gene driven by the double CaMV35S promoter (2 × CaMV35S) was used for monitoring transformation events. The full-length coding sequence (CDS) of *Cs2g22100* was amplified from sweet orange (*Citrus sinensis*) genomic DNA and cloned into pNmGFPer to generate the overexpression construct. The primers used for vector construction are listed in Supplementary Table S4. The resulting overexpression construct was verified by sequencing and sequence alignment before introduction into *A. rhizogenes*. Both the empty vector and the verified overexpression construct were separately introduced into *A. rhizogenes* strain K599 by heat-shock transformation using competent cells from Weidi Biotechnology (Shanghai, China), following the manufacturer’s instructions. A single transformed colony was cultured in TY liquid medium supplemented with 50 mg/L kanamycin, 100 mg/L streptomycin sulfate, and 1 mM CaCl_2_ at 28 ± 2 °C with shaking at 200 rpm until the culture reached an OD_600_ of 0.6~0.8.

### 4.3. Explant Preparation and A. rhizogenes-Mediated Transformation

#### 4.3.1. Preparation of Cotyledon and Epicotyl Explants

‘Carrizo’ citrange seedlings were used for explant preparation after the growth period described above. Cotyledon and epicotyl explants were excised from sterile seedlings. For cotyledon explants, cotyledons were excised near the hypocotyl junction, and an additional incision (~0.1 cm) was made on the opposite side of the excised surface to increase the contact area with the bacterial suspension. For epicotyl explants, segments were excised and cut diagonally at approximately 45° to enlarge the infection surface.

#### 4.3.2. Preparation of *A. rhizogenes* Inoculum and Infection

The *A. rhizogenes*-mediated transformation procedure was performed with modifications based on previously described protocols [9]. Briefly, bacterial cells were collected by centrifugation and resuspended in ½ MS liquid medium containing 50 μM AS for induction before infection. For cotyledon transformation, the bacterial suspension was pre-induced with AS for 3 h at 28 ± 2 °C in darkness with shaking at 160 rpm. Cotyledon explants were subsequently immersed in the pre-induced bacterial suspension for an infection duration of 30 min with gentle shaking. When the same conditions were applied to epicotyl explants, excessive bacterial proliferation, tissue browning, and poor recovery of transformed hairy roots were observed during subsequent culture. Therefore, the infection procedure for epicotyl explants was adjusted based on the previously reported citrus epicotyl transformation protocol [9]. Specifically, the AS pre-induction duration was shortened to 1 h, whereas the infection duration was maintained at 30 min with gentle shaking. After infection, excess bacterial suspension was removed using sterile filter paper, and the explants were transferred to hairy root co-cultivation medium (HRCCM). The explants were incubated in darkness at 22 ± 2 °C overnight and subsequently cultured at 25 ± 2 °C for an additional 2 d before transfer to selection medium.

#### 4.3.3. Selection and Hairy Root Induction

Following co-cultivation, cotyledon explants were directly transferred to ½ MS hairy root selection medium for cotyledon (HRSM-C), whereas epicotyl explants were rinsed three times with sterile distilled water, blotted dry with sterile filter paper, and transferred to ½ MS hairy root selection medium for epicotyl (HRSM-E) (Table 2).

Explants were cultured in darkness at 28 ± 2 °C for 7 days, followed by incubation under a 16 h light/8 h dark photoperiod for 3 days. After selection, cotyledon explants were transferred to MS hairy root development medium for cotyledon (HRDM-C), whereas epicotyl explants were washed three times with sterile distilled water, dried with sterile filter paper, and transferred to MS hairy root development medium for epicotyl (HRDM-E). Explants were maintained under a 16 h light/8 h dark photoperiod until hairy roots reached approximately 3~5 cm in length and were subsequently subjected to fluorescence screening.

### 4.4. Screening and Preparation of Transgenic Hairy Roots for Regeneration

Hairy roots were screened for GFP fluorescence using a handheld fluorescence UV lamp. GFP-positive hairy roots with strong fluorescence signals and vigorous growth were selected for subsequent regeneration experiments. Selected hairy roots were excised into approximately 1.0 cm segments and treated with 640 mg/L timentin solution for 30 min to eliminate residual *A. rhizogenes*. The root segments were subsequently rinsed twice with sterile distilled water and transferred to callus induction medium (CIM) to promote root swelling and callus formation.

After 7~10 days of culture, GFP-positive callus tissues derived from hairy root segments were transferred to shoot induction medium (SIM) for adventitious shoot induction. Following shoot emergence and leaf expansion, regenerated shoots were transferred to shoot elongation medium (SEM) until sufficient stem length was achieved for grafting.

Elongated shoots were excised and micrografted onto etiolated ‘Carrizo’ citrange seedlings approximately 2 weeks old. The upper portion of each seedling was removed, leaving approximately 5 cm of hypocotyl with the root system intact. A central slit was made at the top of the remaining hypocotyl. The base of each scion was cut into a wedge and inserted into the slit, and the graft union was wrapped with sterile parafilm. The roots of the grafted seedlings were immersed in MS liquid medium (pH 5.8), with the graft union maintained above the medium surface. Attempts to induce rooting in excised, elongated shoots were unsuccessful under the conditions tested. Micrografting was therefore used to facilitate plant recovery and reduce the loss of GFP-positive shoots during prolonged in vitro culture. Successfully grafted shoots were maintained under controlled growth conditions at 28 ± 2 °C under a 16 h light/8 h dark photoperiod until stable graft unions and sustained scion growth were established. The recovered scions were subsequently excised and regrafted onto larger soil-grown ‘Carrizo’ citrange seedlings for further plant development and establishment.

### 4.5. Molecular Identification of Transgenic Plants

Regenerated plants were initially screened for GFP fluorescence using a handheld fluorescence UV lamp, and GFP-positive plants were selected for subsequent molecular analysis. Genomic DNA was extracted from newly developed scion leaves after micrografting and used for PCR detection of the *Cs2g22100* expression cassette. The forward primer was located within the CaMV35S promoter, and the reverse primer within the *Cs2g22100* coding sequence, allowing amplification across the promoter–gene junction. PCR products were separated by agarose gel electrophoresis. For gene expression analysis, total RNA was extracted from GFP-positive plants and reverse-transcribed into cDNA. Quantitative real-time PCR (RT-qPCR) was performed to evaluate the expression level of *Cs2g22100*, using *CitActin* as the reference gene. Relative transcript abundance was calculated using the 2^−ΔΔCt^ method. The primers used for PCR and RT-qPCR are listed in Supplementary Table S4.

### 4.6. Statistical Analysis

All experiments were performed using at least three independent replicates. Hairy root induction efficiency was calculated as the percentage of explants producing hairy roots. GFP-positive hairy root frequency was calculated as the percentage of GFP-positive hairy roots among the total number of hairy roots analyzed. Shoot regeneration frequency was calculated as the percentage of hairy root explants producing regenerated shoots. Hairy root transformation efficiency was calculated as the percentage of GFP-positive hairy roots relative to the total number of explants.

Statistical analyses were performed using IBM SPSS Statistics version 25. Data were analyzed using one-way analysis of variance (ANOVA), followed by Duncan’s multiple range test for mean comparisons. Results are presented as the mean ± standard deviation (SD), and different lowercase letters indicate statistically significant differences among treatments according to the post hoc test (*p* < 0.05).

## 5. Conclusions

In this study, we established an integrated *A. rhizogenes*-mediated transformation and plant regeneration pipeline for ‘Carrizo’ citrange. Cotyledon and epicotyl explants provided complementary sources of transformed hairy roots, expanding the availability of transgenic root materials for subsequent regeneration. Optimization of AS pre-induction time, infection time, and 2,4-D concentration improved hairy root induction and transformation performance while maintaining normal explant development. Furthermore, transgenic hairy roots were successfully regenerated into shoots through a callus-mediated pathway, and stable transgenic plants were recovered through graft-assisted plant establishment. This integrated pipeline bridges rapid hairy root transformation with whole-plant recovery and provides a practical platform for stable genetic transformation and functional genomic studies in citrus.

## Figures and Tables

**Table 1:** Comparison of *A. rhizogenes*-mediated hairy root induction efficiency between cotyledon and epicotyl explants of ‘Carrizo’ citrange under optimized conditions.

Explant Type	No. of Explants	No. of Explants Producing Hairy Roots	No. of GFP-Positive Hairy Roots	Hairy Root Induction (%)	GFP-Positive Hairy Root Frequency Rate (%)	Hairy Root Transformation Efficiency (%)
Epicotyl 1	400	45	30	11.25	66.67	7.50
Epicotyl 2	625	108	58	17.28	53.70	9.28
Epicotyl 3	450	73	45	16.22	61.64	10.00
Cotyledon1	519	378	278	72.83	73.54	53.56
Cotyledon2	490	330	249	67.35	75.45	50.82
Cotyledon3	483	326	240	67.49	73.62	49.69

Note: Hairy root induction rate (%) = the number of explants producing hairy roots/the total number of explants × 100%. GFP-positive hairy root frequency (%) = the number of GFP-positive hairy roots/the number of explants producing hairy roots × 100%. Hairy root transformation efficiency (%) = the number of GFP-positive hairy roots/the total number of explants × 100%. Each row represents one independent replicate, with three replicates per explant type.

**Table 2:** Media compositions and culture conditions used for *Agrobacterium rhizogenes*-mediated hairy root induction and root-to-shoot regeneration in ‘Carrizo’ citrange.

Medium	Abbreviation	Composition (1 L Volume)	Culture Period
½ MS Liquid Medium	½ MSLIQ	MS Basal Salts (2.2 g), sucrose (25.0 g), MS vitamins (1 mL, 1000×), pH 5.2	—
½ MS Hairy Root Co-Cultivation Medium	HRCCM	MS Basal Salts (2.2 g), sucrose (25.0 g), MS vitamins (1 mL, 1000×), pH 5.8, Gelrite (2.5 g), Acetosyringone (100 µM), Lipoic Acid 50 µM)	Overnight + 2 days
½ MS Hairy Root Selection Medium for Cotyledon	HRSM-C	MS Basal Salts (2.2 g), sucrose (25.0 g), MS vitamins (1 mL, 1000×), pH 5.8, Phytagel (3.0 g), Kanamycin (25 mg), timentin (320 mg), 2,4-D (0.1 mg)	Dark culture for 7 d, followed by light culture for 3 d
½ MS Hairy Root Selection Medium for Epicotyl	HRSM-E	MS Basal Salts (2.2 g), sucrose (25.0 g), MS vitamins (1 mL, 1000×), pH 5.8, Gelrite (2.5 g), Kanamycin (25 mg), timentin (320 mg), 2,4-D (0 mg)	Dark culture for 7 d, followed by light culture for 3 d
MS Hairy Root Development Medium for Cotyledon	HRDM-C	MS Basal Salts (4.33 g), sucrose (30.0 g), pH 5.7, Phytagel (3.0 g), IBA (2 mg), K-NAA (0.5 mg), Kanamycin (25 mg), timentin (320 mg), activated carbon (0.5 g)	Hairy root emergence within approximately 15 d
MS Hairy Root Development Medium for Epicotyl	HRDM-E	MS Basal Salts (4.33 g), sucrose (30.0 g), pH 5.7, Gelrite (2.5 g), IBA (2 mg), K-NAA (0.5 mg), Kanamycin (25 mg), timentin (320 mg), activated carbon (0.5 g)	Hairy root emergence within approximately 6–8 weeks
Callus Induction Medium	CIM	DKW Basal Salts (5.22 g), sucrose (30.0 g), B5 vitamin (1 mL, 1000×), pH 5.8, Agar (8.0 g), 6-BA (0.75 mg), TDZ (0.015), K-NAA (0.05 mg), Kanamycin (25 mg), timentin (320 mg), activated carbon (0.5 g)	Swollen callus formation within 10–15 d
Shoot Induction Medium	SIM	DKW Basal Salts (5.22 g), sucrose (30.0 g), B5 vitamin (1 mL, 1000×), pH 5.8, Agar (8.0 g), 6-BA (2 mg), Kanamycin (25 mg), timentin (320 mg), activated carbon (0.5 g)	Shoot emergence within approximately 4–6 weeks
Shoot Elongation Media	SEM	DKW Basal Salts (5.22 g), sucrose (30.0 g), B5 vitamins (1 mL, 1000×), GA_3_ (0.3 mg), 6-BA (0.05 mg), pH 5.8, Gelrite (2.5 g), carbenicillin (100 mg), cefotaxime (200 mg), activated carbon (0.5 g)	Culture for approximately 7 d

Note: MS, Murashige and Skoog; DKW, Driver and Kuniyuki Walnut. MS basal salts (Coolaber, China) contained ferrous sulfate but no vitamins. DKW basal salts were purchased from PhytoTech Labs (D190, USA). B5 Vitamin (1000×): Coolaber, PML1730-100 mL, MS Vitamins (1000×): Coolaber, PML 1780-100 mL: 2,4-D: 2,4-Dichlorophenoxyacetic acid; IBA; Indole-3 butyric acid; K-NAA: Naphthalene acetic acid potassium; 6-BA: 6-Benzylaminopurine; TDZ: Thidiazuron; GA_3_: Gibberellic acid. Where indicated, vitamin stock solutions were added at 1 mL per liter of final medium to obtain a final strength of 1×.

## Data Availability

Data will be made available by the authors upon request.

## Supplementary Materials

The following supporting information can be downloaded at https://www.mdpi.com/article/10.3390/plants15213410/s1. Table S1: Effect of different acetosyringone (AS) induction times on hairy root induction, GFP-positive rate, and hairy root transformation efficiency in ‘Carrizo’ citrange. Table S2: Effect of different *Agrobacterium rhizogenes* infection times on hairy root induction, GFP-positive rate, and hairy root transformation efficiency in ‘Carrizo’ citrange. Table S3: Effect of different 2,4-D concentrations on hairy root induction, GFP-positive rate, and hairy root transformation efficiency in ‘Carrizo’ citrange. Table S4: Primer sequences used for PCR and quantitative real-time PCR analysis of *Cs2g22100* expression.
