# Supplementary material for: A Cotyledon-Derived Agrobacterium rhizogenes-Mediated Transformation System for Regenerating Transgenic ‘Carrizo’ Citrange Plants
Source: Plants (Basel). 2026 Nov 2;15(21):3410. doi: 10.3390/plants15213410 (PMC13765563; doi:10.3390/plants15213410)
Supplement: Supplementary file 1 [file plants-15-03410-s001.zip › plants-4536730-supplementary.pdf]

Table S1. Effect of different acetosyringone (AS) pre-induction time on hairy root induction, GFP-positive rate, and transformation efficiency in ‘Carrizo’ citrange.

AS induction time (h)	No. of cotyledons	No. of hairy roots	No. of GFP-positive hairy roots	Hairy root induction rate (%)	GFP-positive hairy root frequency (%)	Hairy-root transformation efficiency (%)
1	60	22	8	36.67	36.36	13.33
1	35	16	6	45.71	37.5	17.14
1	60	31	9	51.67	29.03	15.00
2	55	23	11	41.82	47.83	20.00
2	55	21	10	38.18	47.62	18.18
2	40	16	8	40.00	50.00	20.00
3	90	52	24	57.78	46.15	26.67
3	78	48	27	61.54	56.25	34.62
3	60	45	20	75.00	44.44	33.33
4	48	22	12	45.83	54.55	25.00
4	60	22	11	36.67	50.00	18.33
4	60	18	8	30.00	44.44	13.33

Note: Hairy root induction rate (%) = the number of explants producing hairy roots /the total number of infected cotyledon explants×100%. GFP-positive hairy root frequency (%) = the number of GFP-positive hairy roots / the total number of hairy roots×100%. Hairy-root transformation efficiency (%) = the number of GFP-positive hairy roots / the total number of infected cotyledon explants×100%. Each row represents one independent replicate, with three independent replicates per treatment.

Table S2. Effect of different *Agrobacterium rhizogenes* infection times on hairy root induction, GFP-positive rate, and transformation efficiency in ‘Carrizo’ citrange.

Infection time (min)	No. of cotyledons	No. of hairy roots	No. of GFP-positive hairy roots	Hairy root induction rate (%)	GFP-positive hairy root frequency (%)	Hairy-root transformation efficiency (%)
10	48	21	7	43.75	33.33	14.58
10	60	28	9	46.67	32.14	15.00
10	40	18	8	45.00	44.44	20.00
20	42	20	8	47.62	40.00	19.05
20	56	32	14	57.14	43.75	25.00
20	48	24	8	50.00	33.33	16.67
30	90	59	28	65.56	47.46	31.11
30	75	44	26	58.67	59.09	34.67
30	70	42	22	60.00	52.38	31.43
40	55	30	12	54.55	40.00	21.82
40	48	28	10	58.33	35.71	20.83

40	40	25	9	62.50	36.00	22.50

Note: Hairy root induction rate (%) = the number of explants producing hairy roots /the total number of infected cotyledon explants×100%. GFP-positive hairy root frequency (%) = the number of GFP-positive hairy roots / the total number of hairy roots×100%. Hairy-root transformation efficiency (%) = the number of GFP-positive hairy roots / the total number of infected cotyledon explants×100%. Each row represents one independent replicate, with three independent replicates per treatment.

Table S3. Effect of different 2,4-D concentrations on hairy root induction, GFP-positive rate, and transformation efficiency in 'Carrizo' citrange.

2,4-D concentration (mg/L)	No. of cotyledons	No. of hairy roots	No. of GFP-positive hairy roots	Hairy root induction rate (%)	GFP-positive hairy root frequency (%)	Hairy-root transformation efficiency (%)
0	90	15	9	16.67	60.0	10.00
0	80	11	7	13.75	63.6	8.75
0	100	18	14	18.00	77.8	14.00
0.1	50	36	26	72.00	72.22	52.00
0.1	56	35	24	62.50	68.57	42.86
0.1	60	42	29	70.00	69.05	48.33
0.2	60	26	16	43.33	61.54	26.67
0.2	70	30	21	42.86	70.00	30.00
0.2	60	20	13	33.33	65.00	21.67
0.3	60	27	9	45.00	33.33	15.00
0.3	60	31	13	51.67	41.94	21.67
0.3	60	26	10	43.33	38.46	16.67
0.4	60	30	10	50.00	33.33	16.67
0.4	60	26	15	43.33	57.69	25.00
0.4	60	32	11	53.33	34.38	18.33
0.5	90	60	18	66.67	30.00	20.00
0.5	80	51	20	63.75	39.22	25.00
0.5	60	45	14	75.00	31.11	23.33

Note: Hairy root induction rate (%) = the number of explants producing hairy roots /the total number of infected cotyledon explants×100%. GFP-positive hairy root frequency (%) = the number of GFP-positive hairy roots / the total number of hairy roots×100%. Hairy-root transformation efficiency (%) = the number of GFP-positive hairy roots / the total number of infected cotyledon explants×100%. Each row represents one independent replicate, with three independent replicates per treatment.

Table S4 Summary of the primers used in this study.

Primer name	Sequence (5′–3′)	Application
Cs2g22100-TYCZ-F	AGGACAGGGTACCCGGGGATCC ATGGAATTAAAATTAGCTTCCCTCG	Vector construction
Cs2g22100-TYCZ-R	CAGGGAATTCCTGCAGGTCGACTTATTGTTCGATAGGGA TAGGCTC	Vector construction
Cs2g22100-YXJC-F	GGAAAGGCCATCGTTGAAG	PCR
Cs2g22100-YXJC-R	GCTTGCAAAGGTTTAGTGGAG	PCR
Cs2g22100-qPCR-F	GCCGGCCGATTAGAGAAGTT	RT-qPCR
Cs2g22100-qPCR-R	TGATAGGCCACTCCCAACCT	RT-qPCR
CitActin-F	CATCCCTCAGCACCTTCC	RT-qPCR
CitActin-R	CCAACCTTAGCACTTCTCC	RT-qPCR
